# The Promising Role of Polyphenols in Skin Disorders

**DOI:** 10.3390/molecules29040865

**Published:** 2024-02-15

**Authors:** Mohd Farhan

**Affiliations:** 1 Department of Chemistry, College of Science, King Faisal University, Al Ahsa 31982, Saudi Arabia; mfarhan@kfu.edu.sa; 2 Department of Basic Sciences, Preparatory Year, King Faisal University, Al Ahsa 31982, Saudi Arabia

**Keywords:** skin disorder, polyphenols, human health, clinical trials, nanoformulations

## Abstract

The biochemical characteristics of polyphenols contribute to their numerous advantageous impacts on human health. The existing research suggests that plant phenolics, whether consumed orally or applied directly to the skin, can be beneficial in alleviating symptoms and avoiding the development of many skin disorders. Phenolic compounds, which are both harmless and naturally present, exhibit significant potential in terms of counteracting the effects of skin damage, aging, diseases, wounds, and burns. Moreover, polyphenols play a preventive role and possess the ability to delay the progression of several skin disorders, ranging from small and discomforting to severe and potentially life-threatening ones. This article provides a concise overview of recent research on the potential therapeutic application of polyphenols for skin conditions. It specifically highlights studies that have investigated clinical trials and the use of polyphenol-based nanoformulations for the treatment of different skin ailments.

## 1. Introduction

Skin is the biggest organ in the human body, making up around 10–15% of the overall weight [1]. The skin performs multiple activities, such as safeguarding the body, eliminating waste, defending against diseases, maintaining body temperature, and sensing external stimuli [2]. The exacerbation of environmental degradation and depletion of the ozone layer has resulted in an increase in ultraviolet (UV) radiation in the atmosphere. As a result, the chances of acquiring skin cancer, dermatitis, and skin cellular senescence have also increased. External stimuli activate the skin, leading to oxidative stress [3,4,5]. Oxidative stress occurs when there is an overproduction of reactive oxygen species (ROS) in living organisms, which cannot be completely eradicated by the antioxidant system, resulting in an imbalance in redox homeostasis [6]. An overabundance of ROS can directly inflict injury on lipids, proteins, and DNA, leading to oxidative damage to the skin. The interplay between ROS and DNA initiates the activation of proto-oncogenes. ROS induces collagen breakdown by binding to proteins. Likewise, when ROS attaches to lipids, it results in lipid peroxidation and a rise in the permeability of the cell membrane [6]. UV light causes oxidative damage to large molecules, leading to inflammation, accelerated aging, and many types of skin cancer [7]. On the other hand, an excessive amount of ROS is involved in cellular signaling pathways, altering the function of multiple genes and leading to the aging of skin cells, inflammatory skin conditions like psoriasis and dermatitis, and the formation of cancerous growths such as melanoma and squamous cell carcinoma [1,7].

**Figure 1:**
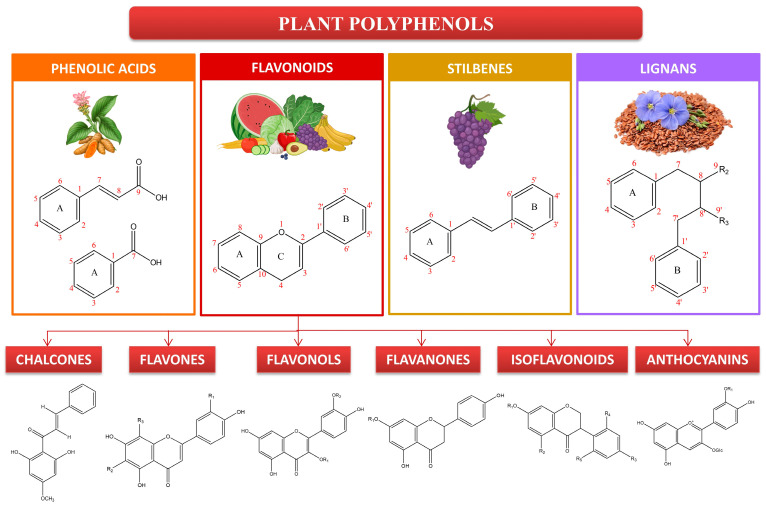
Polyphenols are classified as phenolic acids, flavonoids, stilbenes, and lignans based on their structure and the numbering of carbons.

**Figure 2:**
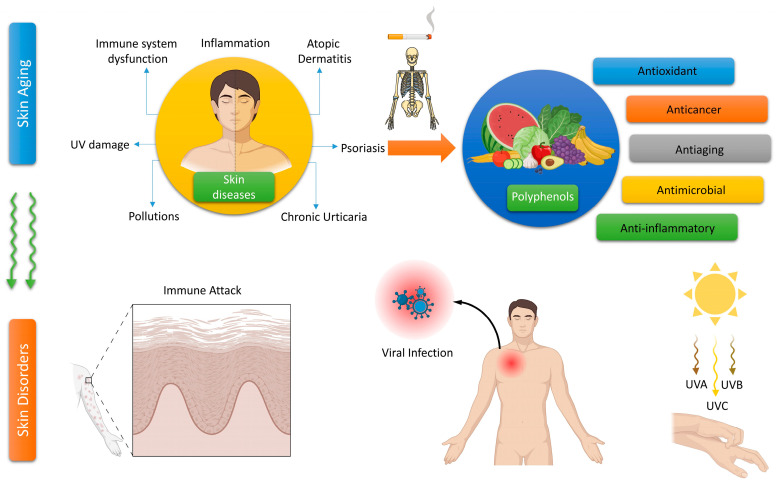
The versatile use of multifaceted polyphenols in treating various skin conditions.

Currently, there are several antioxidants that can effectively address skin cellular senescence, inflammation, and cancer resulting from oxidative stress. Biological antioxidants are chemicals that can effectively delay or prevent the oxidation of a material that is capable of being oxidized, especially when the concentration of the antioxidant is lower than that of the substance being oxidized [8]. The human body is equipped with an antioxidant system comprised mostly of antioxidant enzymes and non-enzymatic antioxidants. These components collaborate to safeguard the human skin from oxidative damage induced by ROS [9]. Antioxidant enzymes include superoxide dismutase (SOD), catalase (CAT), glutathione peroxidase (GSH-Px), glutathione reductase (GSH-Re), and other enzymes [10]. Non-enzymatic antioxidants primarily include diminutive molecules that possess the capacity to neutralize free radicals. This encompasses antioxidant vitamins and their derivatives, cofactors, melatonin, minerals, sulfur compounds, non-protein nitrogen compounds, and plant antioxidants [11,12,13,14,15]. Plant polyphenols are a group of powerful antioxidants present in various biological systems, including red blood cells. Polyphenols are chemicals that originate only from the shikimate/phenylpropanoid and/or the polyketide pathway. They consist of several phenolic units and do not contain any nitrogen-based groups. Polyphenols encompass a wide range of compounds, such as phenolic acids, flavonoids, stilbenes, and lignans (Figure 1). Certain compounds are accountable for the fragrance, pigmentation, and antioxidant characteristics of the fruits, vegetables, seeds, and nuts that we ingest. Polyphenols are gaining significance, mostly due to their advantageous impact on health. The presence of the ortho-phenolic hydroxyl group in the phenolic hydroxyl structure allows for easy oxidation, leading to the creation of a quinone structure. This structure demonstrates a notable ability to remove free radicals, particularly ROS [16,17]. Moreover, these polyphenols possess the capacity to activate cellular signaling pathways and trigger the production of antioxidant enzymes. Plant polyphenols have low aqueous solubility, are easily absorbed, and have limited bioavailability. Currently, there have been many investigations into the ingestion of plant polyphenols, but there is a dearth of adequate clinical research on the application of plant polyphenols topically to the skin [17]. The creation of biomaterials allows for the reliable and effective transport of plant polyphenols to the skin, thus addressing the constraints posed by restricted biocompatibility. This article provides a thorough examination of the precise objectives of plant polyphenols in the prevention of oxidative stress-induced skin cellular senescence, inflammation, and cancer (Figure 2). Furthermore, it explores the latest developments in the delivery systems of plant polyphenols. These data surely serve as a theoretical foundation for future clinical investigations, the advancement of novel pharmaceuticals, and the formulation of cosmetic items.

### Source of the Data

For study selection, the latest versions of databases like Web of Science, Google Scholar, Scopus, and Pubmed were used, along with search keywords such as “polyphenols, flavonoids, and non-flavonoids in skin disorders.” For the literature review, the author looked for the most important and recent studies on chemical and biological properties. The author did this by looking for in vitro, in vivo, and clinical studies. The focus was on more than 240 latest and relevant papers on polyphenols and how they might help prevent and treat skin diseases.

## 2. Polyphenols and Their Importance to Skin Health

### 2.1. The Antioxidant Characteristics of Polyphenols

The imbalance between the body’s antioxidant system and the buildup of free radicals and oxidants is commonly understood as oxidative stress. Many skin diseases, including psoriasis, vitiligo, skin photodamage, skin cancer, systemic sclerosis, chloasma, atopic dermatitis, and pemphigus, are caused by oxidative stress, which damages DNA, lipids, and proteins and activates or inactivates signaling pathways [18]. Some examples of free radicals are ROS, sulfur species (Sul), and carbon species (CO). Superoxide dismutase, catalase, and glutathione peroxidase are enzyme antioxidants, whereas glutathione, ascorbic acid, and tocopherol are examples of non-enzymatic antioxidants [19]. During metabolic processes within cells, ROS such as superoxide anion radical (O_2_^−•^), hydroxyl radical (^•^OH), singlet oxygen (^1^O_2_), hydrogen peroxide (H_2_O_2_), nitric oxide (NO), and peroxynitrite (ONOO^−^) are also generated. There are positive benefits to human health when produced in regulated doses. Several physiological functions, including neurotransmission, gene transcription, and cell signaling, are aided by ROS [20]. The pathophysiology of human skin cancer, skin wrinkling, and dermatologic illnesses are all linked to oxidative stress, which is caused by their increasing concentrations. Inflammatory and allergic skin diseases are influenced by ROS [21]. Produced as waste products of regular mitochondrial metabolism in the processes of the respiratory chain, ROS mostly originate in mammalian cells’ mitochondria [22]. The skin can also produce ROS when exposed to environmental factors like stress, pollution, and UV light. 

To prevent oxidative damage to the skin, the antioxidant defense mechanism is important [23]. Free radicals and radical reactions can be countered by the skin’s built-in defense mechanisms [24,25]. The dermis has a lower content of antioxidants compared to the epidermis [26]. Antioxidants such as GSH, ascorbic acid, uric acid, α-tocopherol, squalene, and ubiquinone, which are non-enzymatic and hydrophilic, are found in the stratum corneum [23,24,25]. Lipophilic antioxidants, including α-tocopherol, and enzymes like SOD, CAT, and GPx are found in the separate layers of the epidermis [27,28]. Enzymatic and water-soluble antioxidants such as glutathione, uric acid, and vitamin C are found in the dermis [23].

Combinations of enzymes and non-enzymatic antioxidants, or hydrophobic and hydrophilic antioxidants, are common defensive mechanisms against free radicals. When exposed to oxidizing substances in excess, however, these reactions may not be enough [28,29]. Studies conducted in living organisms have revealed that the human skin’s dermis and epidermis undergo specific alterations in key antioxidant enzymes and components as a result of both intrinsic and photoaging. Research has shown that both photoaged and naturally aged skin exhibit elevated CAT activity and GR, respectively, in their epidermis. In both old and photoaged skin, the concentration of α-tocopherol was noticeably lower in the epidermis, and in both types of skin, the levels of ascorbic acid were lower in the dermis and the epidermis [29].

In the fight against free radical damage to the skin, polyphenols derived from plants are important. Polyphenols have a wide range of biological activities, which are associated with their structural variety. These compounds can help the skin’s defense and regeneration processes while also scavenging free radicals through multiple mechanisms of action [23,30,31,32,33,34]. One of these is the elimination of ROS through direct reactions with free radicals, scavenging, and improved dismutation of free radicals to less reactive compounds. Another mechanism is the inhibition or potentiation of the action of many enzymes, including oxidases, and the increase in the expression of antioxidant proteins like CAT and SOD. A third mechanism is the chelation of pro-oxidative metal ions, such as iron or copper. Lastly, phenolic compounds can enhance the effects of other antioxidants, such as restoring the original form of tocopherols from their radical form or prolonging the action of ascorbic acid [23,34]. Some polyphenols that have been found to have substantial antioxidant properties are kaempferol, ferulic acid, quercetin, catechin, resveratrol, and myricetin [23].

### 2.2. The Anti-Inflammatory Characteristics of Polyphenols

Inflammatory dermatoses represent a wide variety of cutaneous disorders and influence people of all age groups and skin morphologies. Many chronic inflammatory skin conditions, such as atopic dermatitis, psoriasis vulgaris, and lichen planus, have a cyclic pattern of symptoms that vary over time [35]. The etiology of these disorders is complex, involving a synergistic interplay between genetic and environmental factors that influence the disease’s initiation and development. To be more specific, the stratum corneum, which is the outermost layer of the skin, releases chemicals that carry signals. These molecules initiate a sequence of processes in the body known as a cytokine cascade, which subsequently triggers an inflammatory response. The inflammatory response plays a crucial role in the progression of several skin disorders [35]. Glucocorticoids and biological agents are widely applied to treat inflammatory skin diseases through various processes. Nevertheless, systemic corticosteroids and immunosuppressives can only be employed for limited periods of treatment owing to their substantial adverse effects. The adverse effects associated with this include stunted growth, reduction of blood cell production, increased pressure in the eye, high blood pressure, elevated blood sugar levels, weakened bones, muscle weakness, clouding of the lens in the eye, increased vulnerability to infections, and fragile or easily damaged skin [36]. Biological therapies have revolutionized the management of moderate-to-severe inflammatory skin conditions by selectively targeting specific pathways of inflammation, including interleukin (IL)-4, IL-13, IL-31, IL-12/23, IL-17, thymic stromal lymphopoietin (TSLP), and tumor necrosis factor-alpha (TNF-α) [37]. However, these therapies come with unavoidable, inherent side effects. These encompass severe bacterial, viral, and fungal infections, such as hepatitis B virus infection, recurrence of a dormant tuberculosis infection, and a heightened susceptibility to Candida infections. In addition, they have the potential to exacerbate pre-existing inflammatory bowel disease and, in rare instances, trigger the emergence of new ulcerative colitis [38,39,40,41]. 

Many polyphenols, specifically flavonoids, possess potent anti-inflammatory properties and are capable of regulating immunity [42,43,44,45,46]. Extensive studies have been conducted on numerous natural polyphenols to explore their beneficial effects on autoimmune and inflammatory diseases. Specific polyphenols, such as resveratrol, chlorogenic acid, caffeic acid, pelargonin, and ferulic acid, can control the activation of genes that stimulate inflammation and the production of cytokines, thus influencing the composition of immune cells [47,48]. Curcumin, a non-flavonoid compound, was observed to reduce the levels of TNF, IL-1, VCAM-1 (vascular cell adhesion molecule-1), and ICAM-1 (intercellular adhesion molecule-1) in human umbilical vein endothelial cells. In addition, curcumin also inhibited the concentrations of inflammatory mediators such as prostaglandins and leukotrienes. Topical application of green tea polyphenols (GTPs) and epigallocatechin-3-gallate (EGCG) resulted in the inhibition of prostaglandin metabolites, including prostaglandin D2 (PGD2), prostaglandin E2 (PGE2), and prostaglandin F2α (PGF2α) [49]. Resveratrol can induce the synthesis of endothelial nitric oxide synthase (eNOS), inhibit the function of cyclooxygenase (COX), and deactivate peroxisome proliferator-activated receptor gamma (PPARγ) in both laboratory (in vitro) and living organism (in vivo) environments [50,51]. In an in vivo study, curcumin demonstrated the capacity to decrease the activity of signal transducer and activator of transcription 3 (STAT3) and nuclear factor kappa-light-chain enhancer of activated B cells (NF-κB). Additionally, it decreased the levels of toll-like receptor-2 (TLR-2) and -4 expression. In addition, curcumin increased the expression of PPARγ. Caffeic acid phenethyl ester suppresses the activation of Toll-like receptor 4 (TLR-4) and NF-κB in macrophages that are activated by lipopolysaccharide (LPS) [52]. 

**Figure 3:**
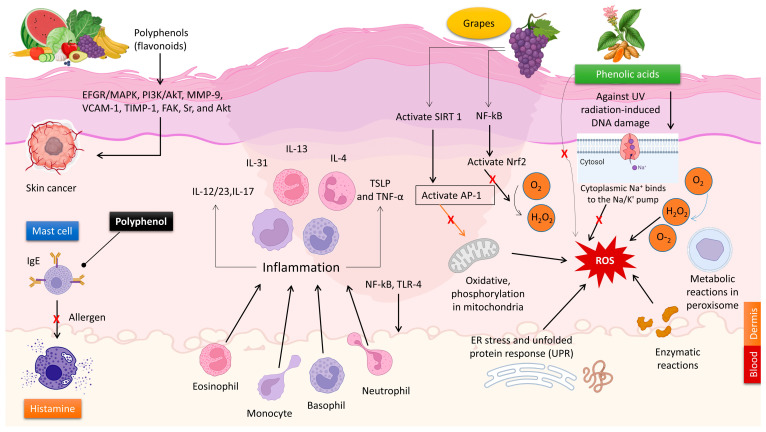
Summary of the effects of polyphenols on skin health and their underlying mechanisms. The anticancer benefits of polyphenols can be attributed to their antioxidant characteristics, along with other associated positive effects.

The anti-inflammatory and immunomodulatory effects of natural polyphenols make them useful in treating various skin disorders. Vitiligo is an inflammatory autoimmune disorder that causes the loss of melanocytes, resulting in depigmentation of the skin [53]. The global prevalence of this condition ranges from 0.5 to 2.0% and exhibits geographical variations. The etiology of vitiligo remains incompletely elucidated [53]. Ginkgo biloba is known for its high concentration of polyphenolic compounds. The topical administration of G. biloba extract was associated with the progression of vitiligo due to its capacity to reduce depigmentation and promote re-pigmentation [54]. Carnosic acid is a naturally occurring abietane diterpene that has a benzenediol structure and is found in rosemary. Carnosol orally significantly reduced the levels of neutrophils, inflammatory cytokines (IL-1β and TNF-α), COX-2, and iNOS in the mice’s circulation [55,56]. Application of carnosol topically demonstrated a considerable reduction in skin lesions in animals afflicted with atopic dermatitis [57]. Artichoke polyphenols possess anti-inflammatory properties and can improve vasodilation and microcirculation in endothelial cells. This is achieved by suppressing the generation of nitric oxide (NO) in both macrophages and endothelial cells. In addition, the polyphenols included in artichoke extract can improve the flexibility and smoothness of the skin by inhibiting the aging process of blood vessels [35]. Artichoke extract can safeguard lymphatic and endothelial cells, making them advantageous. The observed effects can be attributed to the antioxidant or anti-inflammatory properties of the polyphenols, as well as their capacity to indirectly impact molecular pathways (reducing inflammatory markers such as IL-4 and mMCP-8) that promote the activation of genes linked to mechanisms that slow down skin damage (Figure 3) [58].

### 2.3. The Antimicrobial Characteristics of Polyphenols

Antibiotic therapy has been a vital treatment for skin diseases for a considerable period of time. Nevertheless, the medications can result in undesirable side effects and instances of decreased susceptibility to antibiotics. The increasing resistance of germs to synthetic antibiotics has led to a surge in the use of natural products in dermatology. Medicinal plants’ active constituents are currently being explored as potential alternatives for antiseptics and antimicrobials. Flavonoids, such as caffeic acid, benzoic acid, and cinnamic acid, are believed to impact the microorganism by specifically targeting its membrane or cell wall, resulting in both functional and structural harm [59]. Natural polyphenols possess multifaceted characteristics as antimicrobial agents, successfully targeting a wide range of microorganisms, including bacteria, fungi, and viruses. The pomegranate, which originates from the Persian region, contains varying levels of polyphenols at different stages of ripeness. Pomegranate has unique characteristics that render it well-suited for medicinal purposes, specifically in the management of strep throat, hoarseness, and fever. Moreover, it demonstrates antiviral and antiseptic characteristics. The antimicrobial efficacy of pomegranate polyphenols derived from peel, seeds, and leaves was assessed against a variety of microorganisms, such as *Pseudomonas aeruginosa*, *Escherichia coli*, Candida albicans, *methicillin-resistant Staphylococcus aureus* (MRSA), Cryptococcus neoformans, *Mycobacterium intracellulare*, and Aspergillus fumigatus [60]. *Micrococcus luteus* is a nonpathogenic bacteria commonly found on the skin. Nevertheless, it has the potential to transform into a detrimental bacteria and result in grave infections, especially in those with catheters and other pre-existing health issues. The efficacy of pomegranate polyphenols in inhibiting the growth of *M. luteus* was proven by their ability to decrease the production of biofilms. Grape seed polyphenols have exhibited strong antibacterial characteristics and have shown notable efficacy against Gram-positive bacteria, such as *Bacillus cereus*, *Staphylococcus aureus*, *Bacillus coagulans*, and *Bacillus subtilis*. Nevertheless, they have demonstrated superior effectiveness against Gram-negative bacteria, such as *Pseudomonas aeruginosa* or *Escherichia coli* [61]. The investigation revealed that Schinus terebinthifolius exhibited a substantial inhibitory effect on the proliferation of microorganisms, specifically Gram-negative bacteria [62]. Its potential application as a substitute for addressing bacterial resistance can be foreseen. 

A study found 15 clinical *P. aeruginosa* MDR strains. Aztreonam and EGCG synergize in vitro and in vivo since these organisms that are resistant to the antibiotic are vulnerable to the combination. Aztreonam’s antibacterial activity can be restored by EGCG to levels below the EUCAST sensitivity breakpoint for *P. aeruginosa*. EGCG with third-generation cephalosporin cefotaxime synergized [63]. C. longa L. (Zingiberaceae, ginger family), which contains flavonoids and tannins, similarly inhibited clinical *P. aeruginosa* strains in another investigation. The authors found that the ethanolic extract outperformed the plant species’ essential oil against the bacteria tested. They also found a link between ethanolic extract concentration and essential oil: greater extract concentrations reduced bacterial growth by increasing absorbances [64]. Resveratrol showed synergistic activity with azoles on C. albicans strains but no antifungal activity when used alone. The authors found that synergy was demonstrated in over 83% of clinical strains using azoles and resveratrol. It also showed effectiveness against azole-resistant isolates and enhanced the antifungal susceptibility of resistant fluconazole strains. Combining itraconazole and ketoconazole with resveratrol yielded antifungal effects, suggesting that when resveratrol alone is ineffective, a combination with other azole drugs is an alternative strategy [65].

Although current investigations are encouraging, polyphenols need further research to treat and prevent infectious disorders. The development of topical pharmaceutical formulations such as creams, ointments, or lotions with polyphenols to boost clinically utilized antibiotic action to minimize resistance has been investigated. Before using the suggested formulations, it is imperative to engage in clinical studies to evaluate their effectiveness. However, considering the ongoing emergence of resistance and the unclear outlook of existing treatments, undertaking these measures is justified.

### 2.4. The Effects of Polyphenols on Allergic Reactions

Allergic diseases are prevalent in around 40% of the general population and are projected to increase rapidly to 50% [66,67]. Commonly, allergic reactions often impact the skin, resulting in various disorders such as urticaria, angioedema, atopic dermatitis, contact dermatitis, and vasculitis. This phenomenon can be attributed to several cells that have immunological capability, such as mast cells, lymphocytes, eosinophils, neutrophils, and Langerhans cells, specifically the Langerhans cells responsible for antigen presentation [68]. Polyphenols can serve as a substitute for conventional corticosteroid and antihistamine treatments. They possess anti-allergic characteristics, including the capacity to inhibit the synthesis of proinflammatory cytokines and leukocytes, as well as the secretion of histamine [69]. Polyphenols have shown the capacity to regulate the balance between T helper (Th)1/Th2 and impede the generation of antigen-specific IgE antibodies. Polyphenols possess the capacity to impact the process of allergen-IgE complex formation [70]. Moreover, polyphenols possess the capacity to impact the interaction between this chemical and its receptors (FceRI) situated on basophils and mast cells [71]. For instance, research has shown that drinking tannins extracted from apples can help avoid food allergies. This preventive effect may be associated with the increased presence of Gamma delta (γδ) TCR T cells in the intraepithelial lymphocytes of the colon [72]. EGCG significantly hampers the ability of B lymphocytes in peripheral blood to migrate and adhere. The suppressive effect is accomplished by the interaction between EGCG and CD11b receptors on B cells, leading to the prevention of B-cell migration from the bloodstream to adjacent tissues. EGCG exhibits potential as a pharmaceutical agent for the prevention and/or treatment of skin allergy problems, owing to its substantial influence on B lymphocytes, which have a critical function in humoral immunity [73]. To summarize, polyphenols have the potential to be used as anti-allergy medications that can affect several biological processes and immune cell functions associated with the allergic immune response. However, further research is needed to explore this potential.

### 2.5. Skin Cancer Prevention through Polyphenols

The present approach to treating skin cancer, regardless of its metastasis, entails the administration of chemotherapy, immunotherapy, radiation, and targeted therapy. The aforementioned methods are very toxic, expensive, and, in some cases, ineffective due to the development of resistance, especially in metastatic cancer [74]. Therefore, it is essential to propose innovative, efficient treatment methods or drugs that are both economical and more secure. 

Inflammatory mediators promote cancer cell proliferation, invasion, and metastasis [75]. TNF-α participates in inflammatory signaling pathways and contributes to malignant tumor growth [76]. Depending on dose and disease type, TNF-α can cause tumor eradication and progression in cancer patients [77]. Melanoma cells exhibit a considerable increase in the pro-inflammatory IL-6 gene due to TNFα/TNFR1 signaling and TNF-α overexpression [78]. TRADD, an adapter protein linked to the death domain, modulates MAPK and NF-κb pathways via caspase-8 [75,76]. IL-6 overexpression also activates inflammatory signal pathways by causing autophagy and premature senescence [78].

Polyphenols are often linked to TNF-α suppression. Normal human epidermal keratinocytes treated with pomegranate fruit extract before UV exposure showed dose-dependent activation of ERKl/2, JNK1/2, and p38 [79]. In addition, the therapy decreased UV-mediated MAPK phosphorylation over time. Fisetin effectively inhibits melanoma cell invasion by promoting mesenchymal-to-epithelial transition and targeting MAPK and NF-κb pathways in three-dimensionally recreated human skin equivalents [80]. The combination of EGCG and metformin has been shown to suppress NF-κb, p65, and STAT3 signaling pathways in melanoma cells. The polyphenols examined have shown potential as anticancer medicines targeting inflammatory cytokines since they dramatically reduce IL-6, IL-10, and TNF-α, reducing melanoma cell proliferation and migration [81]. Apoptosis, cell migration suppression, cell cycle arrest, and mTOR/PI3K/AKT pathway targeting were shown to be effective anticancer actions of kaempferol on A375 human melanoma cells [82]. Curcumin moderated BK5 IGF-1-induced carcinogenesis. IGF-1 transgenic (Tg) mice by inhibiting IGF-1 receptor, insulin receptor substrate-1, AKT, S6K, and 4EBP1 phosphorylation [83]. Treatment with rosmarinic acid dramatically reduced melanoma cell viability, proliferation, migratory, invasive, and chemotherapy sensitivity by inhibiting ADAM17/EGFR/AKT/GSK3β [84]. Apigenin modulated integrin signaling pathways, caspase-3, FAK/ERK, and cleaved PARP to prevent melanoma cell motility [85]. In a concentration-dependent manner, genistein reduced melanoma cell proliferation and regulated migration and invasion via the FAK/paxillin and MAPK pathways [86]. By downregulating TLR4, tea polyphenols reduce melanoma cell proliferation, migration, and invasion in a dose- and time-dependent manner [87].

Unlike pro-inflammatory cytokines, type I interferon (IFN) activates the immune system to delay cancer [88]. In vitro and in vivo investigations showed that quercetin suppressed melanoma cell proliferation, invasion, and migration by increasing IFN-α and IFN-β expression. Quercetin was thought to activate the retinoic acid-inducible gene I protein (RIG-I) promoter, which increased RIG-I and downstream IRF7. Increased IFN-I upregulated JAK1-STAT1 signaling in a positive feedback loop [89]. Another investigation confirmed contentious STAT1 regulatory findings. In both human and animal melanoma cells, EGCG reduced IFN-γ-induced JAK-STAT activation and PD-L1/PDL2 expression. EGCG also prevented T-cell breakdown and boosted immunological responses (Figure 3) [90].

In addition, matrix metalloproteinases (MMPs), zinc-binding proteases that degrade extracellular matrix (ECM), contribute to malignant cell invasiveness and tumor growth. MMPs are also linked to cellular proliferation, migration, anti- or pro-inflammatory activity, TGF-b and IGF-1 bioavailability, and EMT [91]. Quercetin decreased pro-MMP-9 via the PKC route to prevent murine melanoma B16-BL6 cell invasion [92]. After quercetin therapy, melanoma cells downregulate STAT3-targeted genes MCL-1, MMP-2, MMP-9, and VEGF, which increase cell proliferation, migration, and invasion [93]. Luteolin downregulates MMP-2 and MMP-9 expression via the PI3K/AKT pathway to decrease melanoma cell growth and induce apoptosis [94]. Baicalein also suppressed melanoma cell migration and invasion by inhibiting MMP-2 and -9 expression and activity and the phosphoinositide 3-kinase/AKT signaling pathway [95].

Angiogenesis, which gives nutrition and oxygen to a freshly formed tumor, is a characteristic of cancer. In several tumor progression studies, pro-angiogenic regulators like VEGF-A, bFGF, and Ang1 and Ang2 are upregulated. MMPs degrade the ECM and promote tumor neovascularization. In contrast, angiostatin, TSP-1, TIMPs, and interferons are angiogenic inhibitors. Because varied oxygen levels and oncogene signaling influence angiogenic factor production, several cellular pathways are implicated in angiogenesis [75]. It is reported that resveratrol decreases VEGF and increases TSP-1 (a downstream target of p53) in melanoma-endothelial cell co-culture [96]. Ferulic acid inhibits melanoma development and angiogenesis via the FGFR1-mediated PI3K-AKT signaling pathway in vivo [97]. Resveratrol and 5-FU inhibited cell migration, tumor development, and angiogenesis in B16 murine melanoma cells by downregulating COX-2, VEGF, and VASP [98]. Myricetin therapy in SKH-1 hairless mice effectively reduced UV-induced neovascularization by inhibiting HIF-1α expression and PI-3 kinase activity, which is a critical target for VEGF and MMP suppression [99]. Baicalein and baicalin suppress melanoma cell glucose consumption and metabolism via the mTOR-HIF-1α signaling pathway, resulting in anticancer effects [100]. In vitro and ex vivo angiogenesis experiments showed that naringenin decreases ERK1/2 and JNK MAPK phosphorylation to kill tumor cells and inhibits endothelial cell migration, tube formation, and microvessel sprouting in malignant melanoma [101]. In mouse skin, caffeine inhibited proliferation by modulating the JAK-STAT3 pathway and apoptosis. Caffeic acid also increased TSP-1 expression and protected mouse skin from UV-induced photocarcinogenesis by regulating JAK-STAT3. Chronic UV exposure reduces TSP-1 antiangiogenic protein, which inhibits angiogenesis and proliferation [102]. Delphinidin prevented VEGF-induced proliferation and activation of ERK 1/2 and p38 MAP kinase, a mechanism susceptible to PI3/AKT inhibitors, to diminish B16-F10 melanoma cell xenograft-induced tumor growth [103]. Apigenin stopped melanoma cell motility and invasion by downregulating STAT3 target genes (TWIST1, MMP-2, MMP-9, and VEGF). Apigenin also lowered VEGF expression in melanoma cells, suggesting anti-metastatic effects [104]. Herbacetin at 1 mg/kg effectively inhibited A375 human melanoma xenograft tumor development. Herbacetin also blocked the EGFR signaling pathway, which phosphorylated ERK and AKT to reduce melanoma cell angiogenesis. MMP-9, a pro-angiogenesis factor, was inhibited by herbacetin [105].

As a result, polyphenols could be a viable source of new preventive formulations for melanoma due to their significant anticancer effects. Figure 3 depicts the key mechanisms regulated by polyphenols in skin cancer progression. Table 1 summarizes the studies on polyphenol(s)-dependent regulation of melanoma development.

### 2.6. The Application of Polyphenols in UV Skin Protection

Extended exposure to UV light can harm the skin. The process induces a substantial production of ROS, resulting in skin damage [107,108]. However, other strategies can be employed to protect the skin. Phytochemical substances, specifically phenolics and flavonoids, have shown significant benefits for skin exposed to UV radiation [109,110,111]. Flavonoids exhibit antioxidant characteristics as a result of their capacity to chelate iron, which can harm lipids and proteins in cellular membranes. Additionally, they possess photoprotective properties and are capable of regulating many signaling pathways. For example, they can suppress xanthine oxidase, an enzyme that produces ROS and contributes to oxidative stress [112,113]. Several phenolic compounds have been recognized as effective antioxidant agents for addressing different skin issues, including those resulting from exposure to UV radiation [110]. 

Apigenin, a well-known flavone, exhibits a photoprotective effect against UV radiation on the skin. This flavone compound can be found in a variety of edible medicinal plants and plant-derived beverages, including red wine, beer, and chamomile tea [114,115]. Quercetin, a flavonol compound, can be found in the outer layer of onions, the outer covering of apples, and the foliage of *Hypericum perforatum* L. [116]. Topical use of quercetin effectively protected hairless mice from skin damage induced by UV exposure [117]. Additionally, the extract of *Ginkgo biloba* L. (EGb 761), which has a high concentration of quercetin derivatives, showed the capacity to alleviate symptoms of sunburn in a study involving mice that had UV-induced skin damage. Oral consumption of EGb 761 may have a preventive and therapeutic effect, according to the findings [118]. Silymarin is a highly concentrated extract of flavonolignans derived from the fruits of the milk thistle plant (*Silybum marianum* (L.) Gaernt.). The main constituent of the substance is mostly silybin, which serves as the principal active component [119]. Silymarin, when applied topically in an in vitro study, facilitated the restoration of DNA damage induced by UV radiation. The healing mechanism inhibited apoptosis, or cell death, in both human epidermal keratinocytes and fibroblasts that were subjected to UV light [120]. Genistein, an isoflavone included in soybeans, has been recognized as a photoprotective substance capable of diminishing UV-induced DNA harm in a human skin model that is equivalent in vitro. Genistein’s properties indicate that it may offer protection against photocarcinogenesis. Furthermore, the influence of genistein was examined on UV-induced senescence in human dermal fibroblasts via the oxidative pathway. Genistein was found to efficiently maintain the activities of antioxidant enzymes and control mitochondrial oxidative stress [121,122]. Equol is acknowledged as a byproduct of the isoflavones daidzein or genistein, which are synthesized by the microorganisms present in the gastrointestinal tract [122,123]. An experiment conducted on hairless mice revealed that the topical use of equol before UV radiation exposure can successfully inhibit UV-induced erythema-associated edema, immunosuppression, and skin cancer, which is accomplished by acting as a sunblock and inhibiting DNA photodamage [124]. Furthermore, another research group conducted a study to evaluate the skin-protective properties of used coffee grounds against UV-induced photoaging in hairless mice. The research discovered that the application of extracts from used coffee grounds, which contain flavonoids and caffeine, effectively shielded the mice’s skin by diminishing the activity of matrix metalloproteinases (MMPs) [125]. 

Another study was conducted to investigate the preventive impact of isoflavones, which are included in fermented soymilk products, on skin damage resulting from sunlight exposure in people who have undergone ovariectomy and have no hair. The isoflavones were orally administered for a duration of 28 days. The results indicated that the increased concentrations of isoflavone in the skin and blood of mice successfully eradicated ROS generated by UV light. Furthermore, isoflavone also has estrogenic action, resulting in a defensive impact against UV-induced harm on the skin of the animal model [126].

### 2.7. The Use of Polyphenols in Anti-Aging Cosmetics

The global rise in the average age of the population requires an increased demand for both preventive and therapeutic strategies to tackle age-related diseases. Moreover, there is an increasing demand for cosmetic products that use natural ingredients with potent properties to mitigate the impacts of skin aging. Based on statistics from 2011, 63.8% of the anti-aging cosmetic products sold in Europe were found to contain plant-derived treatments, and the percentage rose to 73.8% in 2018 [127]. Cosmeceuticals formulated with polyphenols or plant extracts enriched with polyphenols have been developed to counteract or delay the process of skin aging [128]. Polyphenols are the active constituents present in the majority of the top 10 botanical species, including Vitis vinifera (vine), Butyrospermum parkii (shea, or Vitellaria paradoxa), Glycine soja (soy), Simmondsia Chinensis (jojoba, or Buxus chinensis), Helianthus annuus (sunflower), Theobroma cacao (cocoa), Calendula officinalis (marigold), Limnanthes alba (meadowfoam), Glycyrrhiza glabra (licorice), and Acacia decurrens (black wattle). These plant-based substances are frequently utilized in skincare products that aim to reduce the signs of aging [129].

In a trial comprising twenty participants, the formulations containing 2% and 3% green tea extracts (GTEs) exhibited substantial efficacy in safeguarding against skin photo-aging and photoimmunology-related consequences. The volunteers underwent repetitive exposure to solar-simulated UV radiation on their upper back, with a dosage of 1.5 minimum erythema [130]. A group conducted a study that revealed that the application of tannase to green tea extract could amplify its revitalizing impact on the skin [131]. The treatment led to elevated levels of gallic acid, epigallocatechin, and epicatechin, which are the components of the extract. The study had a cohort of forty-two Korean female participants who were in good health and aged between 30 and 59 years. A total of 63.60% of females who used tannase-treated GTE for 8 weeks experienced a notable decrease in wrinkles, but only 36.30% of women who used standard GTE reported similar benefits. Over the course of 28 days, a group of 20 healthy women, ranging in age from 36 to 52 years, had a commercial ginkgo preparation called Flavonoids complex SC^®^ applied to their forearms. This treatment led to a 27.88% improvement in skin moisturization, a 4.32% enhancement in skin smoothness, a 0.4% reduction in skin roughness, and a 4.63% decrease in wrinkles. However, a treatment involving a blend containing tea and rooibos (Tealine^®^) has shown the highest efficacy in diminishing wrinkles, resulting in a reduction rate of 9.9% [132]. The skin moisturization was significantly improved by Gingko in comparison to the tea and rooibos blend. The administration of Gingko extract was linked to the advancement of vitiligo through its ability to decrease depigmentation and stimulate repigmentation [35].

Resveratrol, a compound possessing various beneficial biological features such as potent antioxidant, anti-inflammatory, and regulatory actions on Nrf2 and SirT1, also exhibits the capability to inhibit tyrosinase, a crucial enzyme involved in melanin synthesis [133]. A study conducted on 15 people in good health showed that resveratrate, a stable derivative of resveratrol, offered defense against sunburn and suntan resulting from repeated exposure to simulated UV radiation from the sun [134]. An experiment conducted with a group of 20 women revealed that the application of a cream containing 0.8% resveratryl triacetate on their faces twice daily for a duration of 4 and 8 weeks led to notable enhancements when compared to their starting state. The cream decreased the overall surface area of wrinkles by 5.12% and 4.86%, the volume of wrinkles by 10.53% and 8.41%, and reduced sagging by 4.69% and 5.91%. In addition, it resulted in a 2.84% rise in elasticity, a 15.65% increase in denseness, a 5.83% increase in moisture content, a 0.79% increase in lightness, and a 5.43% increase in ITA° (a skin color index) [135]. Furthermore, a study conducted with a group of 20 individuals showed that the application of a concentrated emulsion containing 2% trans-resveratrol (Medskin Solutions Dr. Suwelack AG) for a duration of 8 weeks resulted in noteworthy enhancements in skin elasticity (+5.3%), skin density (+10.7%), reduction in skin roughness (−6.4%), and decrease in skin dispensability (−45.9%) [136]. Eight women, aged 45 to 70, exhibiting evident clinical indications of photoaging on their facial skin, observed a notable decrease in indicators of aging following the utilization of trans-resveratrol in conjunction with beta-cyclodextrin as a carrier. The therapy was administered bi-daily over a period of one month, leading to enhanced brightness, moisturization, and flexibility of the skin [137]. A controlled and randomized trial was undertaken to examine the benefits of applying a serum containing 1% Vitis vinifera shoot extract with a combination of serum and cream to the skin of 60 female participants. The trial spanned a duration of four weeks, during which the participants remained uninformed about the treatment they were assigned to. The study findings revealed a significant improvement in multiple clinical markers of skin aging resulting from sun exposure, including enhanced firmness, radiance, texture, and a reduction in the visibility of fine lines and wrinkles [138]. Topically administering a stable emulsion of 2% Muscat Hamburg grape seed extract in a mixture of water and oil on the cheek skin of male Pakistani volunteers for a duration of 8 weeks led to favorable improvements in facial skin elasticity, sebum content, and melanin levels [139]. In addition, polyphenols help stimulate collagen production, which is essential for skin suppleness and structure [26]. Inhibitors of collagen-degrading enzymes and polyphenols help maintain skin texture and delay the aging process. In a study conducted on human skin cells exposed to UV radiation, it was discovered that quercetin and kaempferol had the ability to decrease the expression of matrix metalloproteinases (MMPs). Skin sagging and wrinkles are caused by MMPs, which are enzymes. The antioxidants quercetin and kaempferol may aid in skin health by decreasing matrix metalloproteinases (MMPs) [75]. 

Hyperpigmentation, a disorder characterized by excessive melanin deposition, can lead to skin darkening and can be caused by various factors. To achieve clinical improvement in pigmentation, one can limit the activation of melanocytes, the synthesis of melanin, or the transfer of melanin. An investigation examined the impacts of ellagic acid, liquiritin, and procyanidin administered by oral and topical routes. Ellagic acid and liquiritin inhibit melanogenesis by chelating copper at the active site of the copper-containing enzyme tyrosinase. The potent antioxidant capacity of procyanidin, which hinders the generation of ROS and the formation of melanin due to UV exposure, may elucidate its efficacy in the treatment of hyperpigmentation [140,141]. Research has also shown that it decreases the activity of NF-κB, an important protein that controls the production of inflammatory substances, and increases melanin synthesis. Current research suggests that polyphenols may be an effective source of skin protection from the effects of UV radiation. Application and consumption of different types of polyphenols that also act as a UV filter have been shown to lead to lower UV-caused skin sunburn [75]. 

## 3. Polyphenols and Their Significance in Skin Disease Therapy

### 3.1. Polyphenols for the Treatment of Vitiligo

Vitiligo is an autoimmune dermatological condition defined by the elimination of melanocytes, leading to depigmentation of the skin and mucous membranes. This results in the progressive expansion of the discolored area of the skin [140,141]. Oxidative stress is a pivotal factor in the initiation and progression of vitiligo. There is significant evidence suggesting that oxidative stress causes dysfunction in melanocyte molecules, exposure to antigens specific to melanocytes, and ultimately, the death of melanocyte cells [142]. The etiology of this disease remains incompletely comprehended; nonetheless, there exists strong evidence suggesting that oxidative stress plays a pivotal role in the pathogenesis of vitiligo. Multiple environmental and internal variables intensify the stress encountered by melanocytes, leading to an excessive generation of hydrogen peroxide (H_2_O_2_). Consequently, oxidative stress is induced, leading to the impairment of Nrf2 signaling activation in vitiligo melanocytes. Membrane peroxidation and the accumulation of ROS peroxidation have been detected in melanocytes and keratinocytes situated in unaffected regions of vitiligo. In addition, the cytokines IL-1, IL-6, and TNF significantly increase the production of IL-8 in melanocytes, resulting in oxidative stress and the programmed cell death of keratinocytes and melanocytes [143]. This disease demonstrates a worldwide occurrence, unaffected by variables such as age and gender. Multiple methodologies are available; however, there is no conclusive solution for vitiligo [144]. The therapeutic efficacy of natural compounds in treating vitiligo is attributed to their capacity to enhance melanin production and impede its breakdown. Such substances primarily function by eliminating free radicals, activating pathways related to melanogenesis, promoting the synthesis of the tyrosinase gene, decreasing the expression of chemokines and inflammatory cytokines, and impeding the migration of CD^8+^ T lymphocytes [142].

Research studies have shown that quercetin has a positive effect on treating pigmentary diseases, both in vivo and in vitro. Quercetin has the ability to protect melanocytes and keratinocytes from oxidative damage. Additionally, the topical administration of quercetin has the potential to protect cells from damage caused by UV light [145]. H_2_O_2_ can lead to the expansion of the endoplasmic reticulum and impede the production of functional tyrosinase in melanocytes, which is a contributing factor in the development of vitiligo. Quercetin can inhibit the series of oxidative reactions regulated by H_2_O_2_, resulting in a decrease in the occurrence of vitiligo [146]. The effect of quercetin on melanin production was investigated in cultured cells, namely HMVII melanoma cells and human epidermal melanocytes (NHEM), using different concentrations (1, 5, 10, 20 μm) and time periods (1, 3, 5, 7 days). Quercetin was found to stimulate melanogenesis by increasing the activity of tyrosinase and decreasing the presence of melanin synthesis inhibitors. There was a noticeable rise in the concentration of melanin, which depended on the dosage and duration [147]. The objective of the study was to evaluate the melanogenic properties of 14 distinct flavonoids and to investigate the correlation between their chemical structures and their influence on melanin synthesis. It has been determined that the hydroxyl group connected to the phenyl ring stimulates melanogenesis. The flavonols quercetin, kaempferol, rhamnetin, and fisetin; the flavones apigenin, luteolin, and chrysin; and the isoflavone genistein were discovered to stimulate melanogenesis. Nevertheless, rutin, myricetin, epigallocatechin gallate, and naringin did not exhibit this particular impact [141]. The presence of eight flavonoids (quercetin, kaempferol, rhamnetin, fisetin, apigenin, luteolin, chrysin, and genistein) resulted in an augmentation of tyrosinase activity in HMVII cells. This effect was achieved by stimulating the production of the tyrosinase protein [148]. There is abundant evidence supporting the advantageous effect of kaempferide, a mono-methoxyflavonol that is produced from kaempferol by adding a methyl group at the 4′-O position. The study showed that kaempferide significantly increased the expression of genes related to the production of melanin (MC1R, MITF, TYR, TYRP1, and DCT) and the activity of the enzyme tyrosinase in the B16F10 melanoma cell line. When exposed to doses of 16–32 μm for a duration of 24 h, Kaempferide did not display any detectable harm to the B16F10 melanoma cell line [149].

### 3.2. Polyphenols for the Treatment of Atopic Dermatitis

Atopic dermatitis is a chronic, inflammatory skin disorder that affects a significant number of individuals worldwide. The precise mechanisms producing atopic dermatitis remain uncertain, while factors such as heredity, immunological responses, and environmental factors have been proposed as potential etiological factors, along with changes in skin barrier function [150]. Recent studies have established a connection between loss-of-function mutations in filaggrin, a crucial protein in the epidermis that maintains the integrity of the skin barrier, and the development of atopic dermatitis [151]. These abnormalities lead to a genetic dysfunction of the epithelial barrier, facilitating the entry of allergens and microbes. This can trigger a disrupted Th2 lymphocyte response, ultimately leading to the development of eczema [152]. Studies have demonstrated that Th2 cytokines have the ability to regulate the production of filaggrin and other crucial structural proteins and peptides, which play a vital role in preserving the integrity of the microbial barrier [153]. While the extent of T cells’ contribution to the altered barrier function in atopic dermatitis is still debated, it is commonly acknowledged that they have a crucial role in the disease’s pathogenic mechanisms. People diagnosed with atopic dermatitis often have a weakened skin barrier function, making them more susceptible to skin damage from environmental factors such as UV radiation. Studies have examined the ability of polyphenols to offer skin protection in individuals with atopic dermatitis [154,155]. A study showcased the impact of apple polyphenols on individuals suffering from atopic dermatitis. The analysis revealed a decrease in inflammation, fissures, pruritus, sleep disturbance, and peripheral blood eosinophil counts among the patients. Furthermore, the research demonstrated that administering individuals with atopic dermatitis a daily dosage of apple condensed tannins at a concentration of 10 mg/kg significantly enhanced their symptoms [156]. 

Resveratrol is an endogenous polyphenol found in several fruits and vegetables, particularly in red grapes and berries. The compound has beneficial properties, including anticancer, antioxidant, antiangiogenic, and anti-inflammatory actions [157,158]. Studies have shown that resveratrol can effectively reduce inflammation and histological changes in a mouse model induced with DNFB. This is achieved by exerting control over apoptosis and modulating the release of cytokines in the epithelium [159]. A study investigated the effects of applying resveratrol-enriched rice (RR) on skin barrier dysfunction and itching in DNCB-treated NC/Nga mice. The study revealed that RR, a genetically modified type of rice that combines the benefits of resveratrol and rice, effectively reduced these symptoms [160]. This finding was accomplished by reducing the production of pro-inflammatory epithelial cytokines, resulting in a reduction in cell toxicity. Notably, out of the several versions that were studied, RR demonstrated the strongest anti-inflammatory, skin healing, and anti-itch characteristics, emphasizing the benefits of resveratrol. According to these data, the authors propose that RR may be a feasible alternative therapy for efficiently addressing the itching and inflammation associated with atopic dermatitis [161]. These findings suggest that polyphenols can protect the skin of people with atopic dermatitis from damage caused by UV radiation and improve the effectiveness of the skin’s protective barrier. However, further studies are necessary to authenticate these findings and ascertain the optimal amount and composition of polyphenols for the purposes of photoprotection and the treatment of atopic dermatitis. 

It is also believed that atopic dermatitis is a complex illness that results from a combination of genetic and environmental factors. The presence of an unequal distribution of Th1 and Th2 cells is the cause of heightened inflammation in atopic dermatitis, emphasizing the crucial involvement of the immune response in the condition’s onset and advancement [161]. Research has been conducted on polyphenols due to their potential to alleviate the symptoms of atopic dermatitis. These compounds possess antioxidant and anti-inflammatory properties that may help modulate the immune response. GTPs have been found to hinder the inflammatory reaction in skin cells and reduce the severity of the symptoms of atopic dermatitis in mice. A study was conducted in which mice suffering from atopic dermatitis were given GTPs. The researchers noted a decline in the synthesis of pro-inflammatory cytokines and chemokines in the skin, accompanied by a decrease in the intensity of atopic dermatitis symptoms [162]. Research has also shown that pomegranate extract reduces the generation of pro-inflammatory cytokines in skin cells and improves the functioning of the skin barrier. This is especially advantageous for those with atopic dermatitis, a disorder characterized by impaired skin barrier function [163,164]. The studies suggest that polyphenols hold promise for treating atopic dermatitis. 

**Figure 4:**
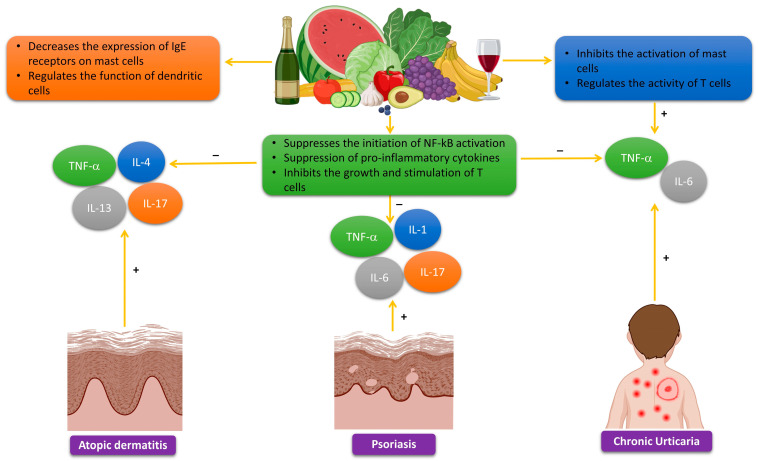
The potential role of polyphenols in the treatment of significant skin disorders. The potential of polyphenols as an additional therapeutic, along with their ability to mitigate deleterious effects, could also improve the negative consequences caused by sunshine exposure.

Polyphenols demonstrate anti-inflammatory effects by inhibiting the production of pro-inflammatory cytokines, such as IL-4, IL-5, and IL-13. Furthermore, research has shown that polyphenols have the ability to reduce the synthesis of IgE receptors in mast cells. Consequently, this results in a decline in the attachment of IgE antibodies, leading to a subsequent reduction in the release of histamine and other substances that cause inflammation. Polyphenols, such as quercetin and EGCG, have shown the capacity to control the functioning of dendritic cells. These cells play a vital role in initiating the Th2 immune response [165,166]. Atopic dermatitis generally ameliorates throughout the summer, and patients can improve the healing of sun-induced skin lesions by utilizing topical therapies that are abundant in polyphenols or by taking nutraceuticals that are based on polyphenols (Figure 4).

### 3.3. Polyphenols for the Treatment of Acne Vulgaris

Acne vulgaris is a common and long-lasting inflammatory skin disorder that mostly impacts the pilosebaceous unit. Acne is a dermatological disorder that impacts both males and females, with a higher prevalence among teenagers and young adults, constituting more than 85% of reported cases [167,168]. Research suggests that acne can persist throughout adulthood. The prevalence of acne is 51% among women aged 20–29 years, but it is 27% among women aged 40–49 years. Acne leads to the formation of lasting scars and psychological issues such as melancholy and anxiety, which negatively impact one’s general health and quality of life [141,169]. The main clinical manifestations of acne lesions include non-inflammatory (open and closed comedones) or inflammatory (papules and pustules) types, primarily occurring on the face, neck, and back. The occurrence of acne is marked by the overgrowth and secretion of sebaceous glands, anomalous production of keratin, inflammation, and the existence of *Propionibacterium acnes* (v) bacteria within hair follicles [170,171]. Acne treatment typically entails the application of benzoyl peroxide, retinoids, antibiotics, or a combination of these substances. Although there is no flawless approach to treating acne, the majority of individuals may discover a suitable regimen to diminish the visibility of imperfections. Moreover, additional investigation is necessary to offer more dependable facts concerning the comparative effectiveness of traditional topical and systemic therapies for acne [172]. Flavonoids were discovered to exert a favorable antibacterial effect on *P. acnes*. The study found that some flavonoids found in licorice, including licochalcone A, licochalcone C, licoflavone A, neobavaisoflavone, liguiritigenin, and isoliquiritigenin, possess anti-acne activities via inhibiting the PI3K-Akt signaling pathways and decreasing mitochondrial activity [173]. 

Quercetin successfully suppressed the production of proinflammatory cytokines in HaCaT, THP-1, and RAW 2647 cell lines that were stimulated by *P. acnes*. Quercetin reduced the amount of TLR-2 protein, the quantity of MMP-9 messenger RNA, and the activation of MAPK via phosphorylation. A study was undertaken on living species, specifically mice, where *P. acnes* was injected intradermally into their ears. Quercetin treatment delivery led to a considerable reduction in ear thickness and swelling [172]. Asposomes, nanovesicles containing quercetin and comprised of ascorbyl palmitate, were developed and utilized for the treatment of acne. Their ability to kill microorganisms was evaluated against *P. acnes* using 3T3 CCL92 cell lines. The asposomes that were synthesized had a size range of 125–184 nm and exhibited stronger antibacterial activity against *P. acnes* when compared to quercetin alone. Quercetin asposomes, when given to a cohort of 20 individuals with acne, successfully reduced the occurrence of inflammatory lesions, comedones, and overall lesions [174]. 

A study was conducted to evaluate the influence of kaempferol and quercetin, both separately and in conjunction with antibiotics erythromycin or clindamycin, on antibiotic-resistant *P. acnes*. The bactericidal properties of quercetin and kaempferol were demonstrated at minimal inhibitory concentrations of ≤32 μg/mL and ≤64 μg/mL, respectively. The research discovered that the pairing of clindamycin with kaempferol or quercetin resulted in a more significant synergistic impact when compared to the pairing of erythromycin with kaempferol or quercetin [175]. A separate study demonstrated the suppressive effect of kaempferol and (+)-catechin on *P. acnes* GehA (glycerol-ester hydrolase A), indicating their potential for acne treatment [128]. 

Utilizing green tea extract, which consists of 57% EGCG and 16% epicatechin gallate (ECG), along with minor quantities of other catechins, led to a decrease in acne blemishes, notably on the nose, perioral area, and chin. The experimental group, including 40 patients, received a daily dose of 1500 mg of green tea extract for a period of four weeks. Each capsule contained 500 mg of the extract. On the other hand, the control group, which also included 40 participants, was administered cellulose as a placebo [176]. The presence of EGCG in human SEB-1 cells resulted in a reduction in sebum production by controlling the AMPK/SREBP-1 signaling pathway. It also controlled inflammation by inhibiting the NF-κB and AP-1 pathways. Furthermore, it facilitated the death of SEB-1 sebocytes through apoptosis and decreased the survival of *P. acne*. An 8-week randomized clinical trial showed that EGCG had a beneficial effect on acne, significantly reducing its occurrence [177]. Research findings indicate that tea polyphenols have demonstrated efficacy in either decreasing sebum production or enhancing acne severity based on at least one measure of outcome.

Nobiletin, a chemical present in citrus fruits, improves the efficacy of acne treatment by suppressing the production of triacylglycerol via diacylglycerol acyltransferase inhibition. Additionally, it inhibits the proliferation of sebocytes and the progressive excretion of sebum. The observed effects are unrelated to cellular demise but rather rely on the translocation of phosphatidylserine to the outer membrane, a process triggered by protein kinase A [141].

### 3.4. Polyphenols in the Treatment of Psoriasis 

Psoriasis is a chronic dermatological illness defined by immune system malfunction, resulting in physical deformity, discomfort, and impairment. The precise etiology of psoriasis remains unclear, while it is thought to be predominantly driven by a confluence of genetic, immunological, and environmental variables with oxidative stress [141,178,179]. Additionally, there is research pertaining to the etiology of psoriasis, indicating that the development of this condition is influenced by both external and internal factors. Various scientific studies have demonstrated that a range of variables, including minor injuries, sunburn, infections, medications affecting the whole body, stress, smoking, air pollution, physical harm, and biological agents like viruses and bacteria, can all contribute to the degradation of keratinocytes [141,180]. Studies have demonstrated that both ROS and NOS (nitric oxide synthase) contribute to the pathogenesis of psoriasis. Hence, an inequilibrium in redox reactions and elevated levels of inducible NOS are responsible for the generation of oxidative stress [181]. Psoriasis can occur at any age; however, it is most frequently seen in people aged 50 to 69 years. Psoriasis has a worldwide impact on about 125 million people, with a prevalence incidence of 2–3% globally [141,182]. Psoriasis vulgaris, the most common form of psoriasis, is characterized by almost symmetrical red and scaly patches and elevated bumps that are coated with white or silver scales. The symptoms are most noticeable on the outside surfaces, scalp, and lower back area [141,182]. The histology findings of psoriasis demonstrate a strong proliferation and atypical differentiation of keratinocytes, together with the infiltration of inflammatory cells [182]. Typical treatments for psoriasis include phototherapy, photochemotherapy, and immunosuppressive drugs such as methotrexate and cyclosporine. The treatment for psoriasis includes the application of topical therapies that comprise salicylic acid, urea, tar, glucocorticosteroids, and vitamin D3 derivatives [183]. Moreover, there is sufficient evidence substantiating the advantageous effect of flavonoids in the management of psoriasis. Currently, the use of prescription medications, following a healthy lifestyle, and consuming a diet rich in antioxidants have the potential to reduce the damaging effects of oxidative stress caused by psoriasis, especially on the skin [184]. The overproliferation of keratinocytes and chronic inflammation in psoriasis are associated with increased concentrations of TNF and vascular endothelial growth factor (VEGF). 

The effects of luteolin on psoriasis lesions were evaluated through research conducted on both normal human epidermal keratinocytes and human keratinocyte (HaCaT) cells. The experiment shows that pre-treatment with luteolin (1–100 μM) significantly inhibits the mRNA expression and release of IL-6, IL-8, and VEGF in a way that depends on the dosage. Luteolin decreases the phosphorylation, movement into the nucleus, and binding to DNA of NF-κB induced by TNF [185]. A separate investigation analyzed the effects of luteolin on BALB/c mice with psoriasis-like lesions generated by imiquimod (IMQ). Luteolin was discovered to improve psoriasis-like skin lesions by inhibiting the infiltration of immune cells and reducing the expression of IL-6, IL-1β, TNF-α, IL-17A, and IL-23. The anti-inflammatory effect was attained by inhibiting the expression and activation of NF-κB. The results also showed that the treatment of luteolin suppressed the synthesis of nitric oxide, iNOS, and COX-2 (Figure 4) [186]. 

Delphinidin, an anthocyanidin, has demonstrated therapeutic effects in the treatment of psoriasis. Delphinidin was administered topically on the skin of female homozygous flaky skin mice aged five weeks at dosages of 0.5 mg/cm^2^ and 1 mg/cm^2^. This application was performed five times per week over a span of 14 weeks. Treatment with delphinidin reduced the levels of pathological markers linked to psoriatic lesions and inhibited inflammation. The study documented the activation of caspase-14, the decrease in infiltrating macrophages, and the downregulation of keratin 14, resulting in hyperproliferation [187]. The effects of delphinidin were evaluated on a three-dimensional reconstructed model of human psoriatic skin, with concentrations ranging from 0 to 20 μM and a duration of 2 to 5 days. The results indicated that delphinidin enhanced the cornification process without affecting apoptosis or the expression of mRNA and proteins for differentiation markers (caspase-14, filaggrin, loricrin, and involucrin). Delphinidin administration led to a decrease in the expression of markers linked to cell proliferation and inflammation [188]. 

Baicalein, a flavone derived from the roots of Scutellaria baicalensis and Scutellaria lateriflora, was topically administered to the psoriatic lesions of BALB/c mice for four consecutive days, following a 5-day treatment with IMQ. Following the administration of baicalein, a significant improvement was observed in the redness, peeling, and thickness of the outer layer of the skin. The levels of IL-17A, IL-22, IL-23, and TNF in the skin showed a significant decrease [189]. Male Balb/c mice were administered S. baicalensis extract, which led to a significant reduction in epidermal thickness and improvement of psoriatic lesions. Furthermore, it hindered the activation and penetration of macrophages by decreasing the levels of inflammatory substances such as NF-κB and COX-2 [190]. It was also discovered that the topical use of astilbin, derived from the rhizome of *Smilax sp*., produced similar results [191]. 

Quercetin demonstrates a significant effect in treating psoriasis. Quercetin, given perorally to mice stimulated with IMQ at dosages of 30, 60, and 120 mg/kg for seven days, caused a notable decrease in TNF-α, IL-6, and IL-17 levels. In addition, it resulted in elevated levels of GSH, CAT, and SOD activities while reducing the accumulation of MDA (malonedialdehyde) in the skin tissue caused by IMQ in mice [192].

### 3.5. Polyphenols in the Treatment of Chronic Urticaria

Chronic urticaria is a dermatological disorder marked by the recurring presence of itchy raised patches on the skin, swelling of the deeper layers of the skin, or both, lasting for more than 6 weeks. The cause of the illness involves the activation of mast cells, leading to the production of histamine and other substances that cause inflammation [193]. Moreover, scientific evidence has shown that oxidative stress and ROS play a significant role in the progression of chronic urticaria [194,195]. There is growing evidence from experiments and clinical studies that indicates an autoimmune origin for many cases of chronic urticaria [196,197]. An estimated 45% of individuals diagnosed with chronic urticaria are thought to have an autoimmune etiology. The intradermal administration of autologous serum, referred to as the autologous serum skin test (ASST), induces a wheal-and-flare response in a significant proportion of individuals with ongoing illness, with prevalence rates ranging from 30% to 60%. Furthermore, the serum obtained from particular individuals with chronic urticaria can stimulate the release of histamine from cultured basophils in healthy people. The presence of these events has been attributed to the existence of circulating IgG antibodies that specifically attach to the high-affinity IgE receptor FceRI, which is found on mast cells and basophils, and additionally, IgE may potentially play a role [198]. The IgG antibodies trigger the usual complement cascade [199]. Upon purifying IgG subclasses, it has been observed that the release of histamine is primarily associated with subclasses 1 and 3 [200]. Other autoantibodies could potentially exert an influential effect on chronic urticaria. A recent in vitro study conducted an extensive investigation and discovered that as many as 65% of patients with chronic urticaria possess autoantibodies targeting CD23, the low-affinity receptor for IgE [201]. This particular autoantibody possesses the capacity to indirectly provoke the secretion of the primary cationic protein from eosinophils, subsequently resulting in the release of granules from mast cells in controlled laboratory settings. The frequent coexistence of chronic urticaria and antithyroid antibodies, both well-established markers of autoimmunity, further supports the autoimmune origin of this disease. Various studies have demonstrated that polyphenols, specifically flavonoids, possess antioxidant properties and can reduce oxidative stress [202,203]. Therefore, polyphenols may help prevent and manage chronic urticaria by reducing oxidative stress and suppressing mast cell activation [204]. 

The use of a resveratrol extract demonstrated promising results in suppressing mast cell activation. The study investigated the influence of resveratrol on the control of mast cell activation mediated by MRGPRX2, as well as the mechanism behind it. Resveratrol inhibited the release of granules from mast cells in a laboratory setting and demonstrated inhibition of extravasation, active systemic anaphylaxis, and degranulation of mast cells in mouse models of pseudo-allergy in vivo. The study presents evidence supporting the potential utilization of resveratrol nutraceuticals in individuals with chronic urticaria [205]. 

A study was conducted on a group of 153 patients who were diagnosed with chronic urticaria. These people adhered to a diet that eliminated pseudoallergens and included a substantial quantity of polyphenols. The findings demonstrated that patients who strictly avoided the identified trigger meals achieved a total cessation of symptoms. Hence, implementing an incremental build-up food challenge (IBUF) technique provides a pragmatic approach for persons suffering from chronic urticaria to achieve enduring alleviation of symptoms through adherence to a straightforward dietary regimen. The findings demonstrated a significant amelioration of symptoms among the participants who strictly followed a diet devoid of pseudoallergens and abundant in polyphenols for a duration of 5 weeks. Moreover, there was a notable enhancement in subjective disruption and general well-being [206]. 

A set of studies analyzed the potential utilization of quercetin and luteolin in the management of several illnesses, including chronic urticaria [207]. The authors highlighted other research that emphasized the antioxidant and anti-inflammatory properties of polyphenols, as well as their potential to reduce oxidative stress and mast cell activation in the skin [208,209]. Furthermore, the scientists suggested that the amalgamation of polyphenols and antihistamines could potentially augment the outcomes for individuals suffering from chronic urticaria [210]. Current evidence suggests that polyphenols can potentially improve the treatment of chronic urticaria by reducing oxidative stress and inflammation in the skin. Nevertheless, further investigation is required to obtain a more profound comprehension of the mechanisms behind these effects and to determine the optimal dosages and compositions of polyphenols for concurrent therapy with chronic urticaria. 

Chronic urticaria arises from the stimulation of mast cells, leading to the synthesis of histamine and other compounds that induce inflammation. Research has investigated the potential of polyphenols to alleviate the symptoms of chronic urticaria by leveraging their anti-inflammatory and antioxidant properties, which may reduce the activation of mast cells. Research has shown that polyphenols have the ability to reduce the severity of hives and decrease the production of inflammatory cytokines [211]. Polyphenols demonstrate anti-inflammatory effects in chronic urticaria by inhibiting the production of pro-inflammatory cytokines, such as IL-6 and IL-8. Furthermore, research has shown that polyphenols can successfully impede the activation of mast cells, which play a vital role in the progression of chronic urticaria. Specific chemicals with intriguing properties have shown the capacity to control the functioning of T cells, which could potentially result in disruptions to the immune system [211]. To summarize, the polyphenols’ ability to modify the immune system presents a potentially successful technique for treating chronic urticaria (Figure 4). Their potential as adjunctive therapy, along with their ability to mitigate inflammation and counteract the detrimental effects of ROS, may contribute to the amelioration of sun-induced damage.

## 4. Assessment of Plant Polyphenols for Skin Disease Therapy

Presently, preclinical studies, particularly those carried out on dermal cells and animal dermal tissues, have shown that plant polyphenols possess the capacity to impede skin cellular senescence, inflammation, and skin cancer. However, the number of human clinical studies conducted is typically insufficient. Table 2 presents a summary of several preclinical studies conducted in laboratory settings (in vitro) and living organisms (in vivo), as well as human clinical trials. The investigations produced the following findings: (1) Plant polyphenols are mostly employed as topical or ingested agents in clinical trials. (2) To ensure the intended therapeutic result, plant polyphenols undergo clinical trials where they are evaluated at high doses and for prolonged periods, which could potentially cause skin, intestinal, and stomach irritation. (3) Plant polyphenols are commonly combined with other compounds in medical settings to increase their bioavailability. (4) It is imperative to develop assessment protocols for clinical outcomes that are more varied and unbiased.

## 5. Utilizing Nano Delivery Systems for the Topical Application of Plant Polyphenols

**Figure 5:**
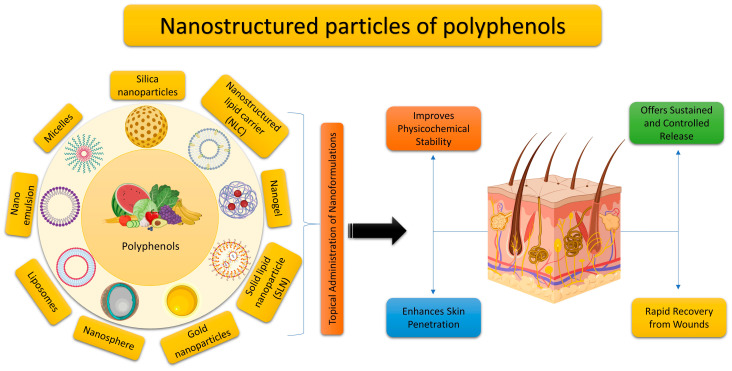
Diverse nanostructured molecules are employed in the creation of nanoformulations for the delivery of polyphenols in skin treatment.

The bioavailability of plant polyphenols is low despite their high bioactivity [17], because most polyphenols are poorly soluble and unstable. The cosmetic and pharmaceutical industries have recently recognized cutaneous delivery as one of the most promising distribution channels. This mode of administration ensures that the polyphenol is continually and steadily released at the site of action, mitigating some of the drawbacks of oral drug delivery, including poor bioavailability, metabolic interactions, and cytotoxicity. But healthy skin has a strong defense against drug penetration, thanks to its unique lipid makeup and stratum corneum tissue. In order to increase polyphenol solubility and bioavailability and provide site-specific drug administration with improved pharmacokinetic properties, the development of nanoengineered polyphenol delivery systems is urgently required (Figure 5). To this day, the most common form of topical administration is gel, especially hydrogels. However, other delivery techniques are now being developed, and recent ones are summarized in Table 3.

## 6. Conclusions and Prospects for the Future 

Polyphenols are widely present in plants and are ingested in significant amounts in the human diet. Polyphenolic (EGCG, resveratrol, quercetin, and curcumin) extracts possess desirable qualities for use in pharmaceuticals and cosmetics, thanks to their advantageous and versatile biological features, as well as their widespread presence in different food sources. Nevertheless, like traditional medications, polyphenols can exhibit toxicity when they exceed permissible thresholds in the human body. Furthermore, certain studies have indicated that polyphenols are ingested through entire foods regularly. Consequently, it remains uncertain whether the observed outcomes are a result of the interactions between polyphenols and other components. Therefore, additional research is required to specifically investigate the isolated forms of polyphenols. Studies examining the positive impacts of polyphenols and their extent must take into account factors such as matrix effects, enzymatic interactions, reactivity with other meals, and genetic or gender traits. Furthermore, the majority of clinical research investigating isolated forms of polyphenols was of short duration. Future studies should focus on elucidating the long-term health benefits and potential detrimental effects. The impact of polyphenols on the skin is mostly dictated by their physicochemical, anti-inflammatory, immunomodulatory, antioxidant properties, and DNA repair activities, which can be exploited for the prevention of a variety of skin disorders. Hence, it is crucial to evaluate the efficacy of polyphenolic substances in treating skin disorders when administered either orally or topically. Although polyphenols possess diverse preventive and therapeutic capabilities and can be utilized for treating various disorders, including those affecting the skin, their efficacy in therapy may be hindered by some limitations, such as their low solubility in water and poor stability and structure. According to reports, the efficacy of polyphenols increases with a greater number of hydroxyl groups. Enhancing the transdermal absorption of plant polyphenols is a prospective area of investigation. Nanostructured particles of polyphenolic-based medications provide numerous benefits compared to conventional drug delivery techniques. Advanced delivery formulations effectively address challenges related to polyphenols’ poor solubility, bioavailability, and stability. Additionally, in the context of topical application, these formulations successfully overcome the significant barrier presented by the skin.

## Figures and Tables

**Table 1:** The impact of polyphenols on the advancement of melanoma tumors (↓ downregulation, ↑ upregulation).

Polyphenol	Study Type	Study Model	Effects	Targets	Ref.
EGCG + metformin	In vitro	B16F10 cells	Inhibition of cell growth and STAT3/NF-κb pathway	STAT3 and NF-κb p65 ↓	[81]
Curcumin	In vivo	BK5.IGF-1 transgenic (Tg) mice	Inhibition of tumor growth	IGF-1 ↓	[83]
Genistein	In vitro	B16F10 melanoma cells	Inhibition of cell proliferation, migration, and metastasis	p-p38, p-ERK, and p-JNK ↓	[86]
Quercetin	In vitro	B16 and A375 cells	Suppressed proliferation	RIG-I ↑ IFN-I ↑ STAT1 ↑	[89]
EGCG	In vivo	C57BL/6 mice	Inhibition of tumor growth	STAT1 ↓	[90]
Rosmarinic acid	In vitro	A375	Inhibits proliferation and migration	ADAM17/EGFR/AKT/GSK3β ↓	[84]
Luteolin	In vitro	A375	Inhibited the proliferation, migration, and invasion	MMP-2 and MMP-9 ↓ TIMP-1 and TIMP-2 ↑	[94]
Apigenin	In vivo	WT mice TKO mice	Inhibition of UV-induced cutaneous angiogenesis	TSP-1 ↑	[106]
Caffeic acid	In vivo	Male Swiss albino mice	Inhibition of angiogenesis and proliferation	TSP-1 ↑	[102]
Resveratrol	In vitro	Co-culture	Antiangiogenic effects	VEGF ↓ TSP-1 ↑	[96]
EGCG	In vitro	1205Lu, HS294T, and A375 cells	Inhibition of cell proliferation	STAT1 ↓	[90]
Herbacetin	In vitro	A375 and Hs294T cells	Suppressed angiogenesis	MMP9 ↓	[105]

**Table 2:** Overview of the studies examining the possible impacts of polyphenols on different skin conditions.

Polyphenol	Type of Skin Cell	Assay	Effect	References
Apigenin	Fibroblasts derived from human foreskin	The cells were co-treated with bleomycin for 24 h at a concentration of either 10 or 20 μM	Downregulated IL-6, IL-8, and IL-1β mRNA expression; Suppressed NF-κB activity	[212]
Kaempferol	Fibroblasts derived from human foreskin	The cells were co-treated with bleomycin for 24 h at a concentration of either 10 or 20 μM	Downregulated expression of IL-6, IL-8, and IL-1β mRNA	[212]
Quercetin	Fibroblasts derived from human foreskin	The cells were co-treated with bleomycin for 24 h at a concentration of either 10 or 20 μM	Downregulated IL-6, IL-8, and IL-1β mRNA expression; decreased SA-β-Gal activity	[212]
Genistein	Co-culture of NHDF with keratinocytes	Applied a concentration of 10 millimolar for a duration of 72 h following exposure to UV radiation	Decreased the production of IL-6; suppressed the phosphorylation of p38, ERK, and JNK	[213]
Gallic acid	NDHF, HaCaT	Applied a concentration ranging from 0.1 to 10 millimolar for a duration of 24 h following UV exposure	Reduced levels of IL-6 and MMP-1; reduced generation of ROS; inhibited phosphorylation of AP-1	[214]
Delphinidin	HaCaT	Used either 5 or 10 micromolar concentration before or after exposure to UV	Restored elastic characteristics	[215]
Fisetin	Senescent mouse embryonic fibroblasts (MEFs) lacking the Ercc1 gene and human IMR-90 fibroblasts	48 h duration of treatment with a concentration range of 1 to 15 micromolar	Decrease in the proportion of cells that are positive for SA-ß-Gal	[216]
Curcumin/ luteolin	MEFs derived from Ercc1 knockout animals	Treatment for 48 h with a concentration of 5 micromolar	Decrease in the proportion of cells that are positive for SA-ß-Gal	[216]
Hawthorn polyphenol extract (HPE)	Human dermal fibroblasts (HDFs) and HaCaT cells were used in conjunction with mice that were 5–6 weeks old.	Cell treatment: HPE (0, 5, 10 µg/mL) for 24 h; mice were administered HPE at doses of 0, 100, and 300 mg/kg.bw.day for a duration of 12 weeks	1. HPE therapy can enhance cell growth, augment intracellular collagen levels, and decrease MMP–1 synthesis 2. Oral HPE mitigates the harmful effects of UV radiation on the skin by removing ROS, decreasing DNA damage, and suppressing the production of p53	[217,218]
Products containing high levels of polyphenols, such as NutroxsunTM, derived from rosemary and citrus	Adult female	For a prolonged period of time, used NutroxsunTM at a dosage of 250 mg per day for two weeks. For a shorter duration, used NutroxsunTM at a dosage of 100 or 250 mg per day for 24 or 48 h	NutroxsunTM, when consumed as part of a diet, decreases the negative effects of UV radiation on the skin, such as wrinkles and loss of suppleness	[219]
Polyphenolic rich extract (SSE and SSW)/Spatholobus Suberectus stem	HaCaT/Human skin	Tg1: The concentration of SSE used was 0, 3, 10, 30, and 300 µg/mL. Tg2: The concentration of SSW used was 0, 3, 10, 30, and 300 µg/mL for a duration of 24 h	1. The presence of SSE and SSW suppressed the generation of ROS and prevented cellular harm. 2. SSE restores skin by increasing the production of enzymes and proteins in cells, preventing the activation of MAPKs phosphorylation caused by UV radiation, and inhibiting the activity of its downstream transcription factor	[220]
Rambutan peel phenolics (RPP)/Nephelium lappaceum; Leu-Ser-Gly-Tyr-Gly-Pro (LSGYGP)/synthetic	Male BALB/c nude mice weighing between 20 and 22 g.	Single group: RPP (100 mg/kg.bw.d), SGYGP (100 mg/kg.bw.d); Composite group: (50 RPP+ 50 LSGYGP) mg/kg.bw.d, (100 RPP + 100 LSGYGP)mg/kg.bw.d/10 weeks	1. The use of RPP and LSGYGP enhanced skin biochemical markers, tissue structure, and collagen levels. 2. RPP improved the control of oxidative stress and the levels of inflammatory factors. 3. The presence of LSGYGP had a substantial impact on the levels of collagen and hyaluronic acid in the skin	[221]
Polyphenols/Flavonoid hesperidin	Hairless male mice that were 6 weeks old.	Water was administered to the control group, while the treatment group received UV radiation along with hesperidin at doses of 0 and 100 mg/kg.bw.d for a duration of 12 weeks	1. Hesperidin, when taken orally, prevented the thickening of the skin and the production of wrinkles caused by UV- radiation. 2. Hesperidin suppressed the UV-induced activation of MMP-9 and cytokines and prevented the degradation of collagen fibers	[222]
Polyphenols/3,5,6,7,8,3,4-heptam-ethoxy flavone (HMF)/C.unshiu peels	HDFn cells (human dermal fibroblast cells)	The samples were treated with different concentrations of HMF (0, 50, 100, 200 µg/mL) for a duration of 24 h	1. HMF shielded HDFn cells from damage caused by UV radiation by suppressing the production of MMP-1 through phosphorylated MAPK signals 2. HMF modulated the expression of Smad3 and Smad7 proteins in a manner that is dependent on the dosage	[223]
Polyphenols/ Tectorigenin/ *Belamcanda chinensis* L.	Human HaCaT cells	Tg refers to the compound Tectorigenin at concentrations of 0, 0.1, 1, and 10 µM. Cg refers to the control group treated with VC at a concentration of 200 µM for a duration of 24 h	1. Tectorigenin reduces ROS levels by enhancing the activity of intracellular antioxidant enzymes. 2. Tectorigenin decreases the expression of mmp–1 and hinders the breakdown of collagen. 3. Tectorigenin exerts an inhibitory effect on apoptosis via modulating the expression of caspase–3 and bcl–2 associated proteins	[224]
Curcumin gel	The skin of breast cancer patients receiving radiation therapy with a range of 36 to 81 years.	Applied the medication topically containing 4% curcumin three times daily for a duration of one week	Topical use of curcumin as a preventive therapy may effectively manage radiation-induced skin inflammation and alleviate associated pain	[225]
Turmeric supplements	Patients between the ages of 18 and 75 with mild scalp psoriasis.	Administered topical cream having turmeric 9% twice a day for a duration of nine weeks	The Dermatology Life Quality Index (DLQI) questionnaire and Psoriasis Area and Severity Index (PASI) scores were evaluated, revealing that the application of turmeric tonic resulted in a considerable reduction in redness, flaking of the scalp and skin abnormalities.	[226]
An anti-itch cream containing a combination of several herbs, with a 16% concentration of turmeric extract and a 0.1% concentration of turmeric oil.	Children aged 2 to 12 with atopic dermatitis	Applied a topical cream containing 16% turmeric twice a day	Both the treatment group, which used an anti-itching cream, and the control group, which used Moisturex, showed significant improvements in all measured aspects, including subjective itching severity, clinical evaluation, and overall health	[227]
Tablets containing pomegranate extract with a high concentration of ellagic acid	Individuals with skin that has undergone aging due to exposure to UV radiation, typically between the ages of 20 and 40.	Administered at a high dose of 200 mg/d ellagic acid or a low dose of 100 mg/d ellagic acid once a day for a duration of four weeks	The questionnaire results indicated that the decrease in skin luminance values was reduced by 1.35% in the low-dose group and by 1.73% in the high-dose group compared to the initial measurement. Furthermore, there was a noticeable upward trajectory in the quality of certain aspects, such as “facial brightness” and “spots and freckles”	[228]
Oral green tea supplement with topical green tea cream	Women having a moderate level of photoaging	Application of a cream containing 10% green tea and taking 300 mg of green tea orally twice a day for a duration of eight weeks	Participants who received a combined treatment of topical and oral green tea demonstrated histological enhancements in elastin levels; however, no noticeable clinical alterations were seen	[229]
Multicomponent polyphenol supplement	Moderate photoaging between the ages of 40 and 65	VitAoX ultra^®^ formula 50 mg *Camellia sinensis* L, two capsules daily for twelve weeks	Increased antioxidant capability and anti-aging metrics	[230]
Taking a vitamin C and green tea supplement orally	Healthy subjects between the ages of 18 and 65	Green tea extract 450 mg per gelatin capsule daily for 12 weeks	UV protection to fibulin-5	[231]
Freshly prepared green tea beverages	Healthy subjects between the ages of 20 and 55	Consumption of 600 mL of freshly brewed green tea beverages per day for a duration of 2 weeks	Enhanced Skin radical scavenging activity	[232]
Tablets containing polyphenols found in apple	Healthy women between the ages of 20 and 39	Tablets of 300 or 600 mg daily for 12 weeks	Prevented UV-induced skin pigmentation	[233]

**Table 3:** Utilizing plant polyphenols in nanotechnology-based skin therapies.

Polyphenol	Delivery Systems	Skin Model	Main Results	Reference
Quercetin	NLC gel	Strat-M membrane and excised rat skin membrane in Franz diffusion cell	There was a notable increase in the ability of the skin to absorb substances when using this gel, as compared to the traditional gel	[234]
	Nanogels	Franz diffusion cell apparatus using Strat-M transdermal diffusion membrane	Chitosan-based nanogel has a lower but controlled skin permeation rate	[235]
	SLN	Vertical Franz diffusion cells, using full-thickness human skin	The quercetin-containing solid lipid-based nanosystems exhibited greater quercetin retention in the skin compared to the control formulation	[236]
	Mesoporous silica	Porcine skin in vertical Franz diffusion cells	Quercetin-loaded mesoporous nanoparticles exhibited enhanced skin accumulation compared to free quercetin	[237]
	Nanoparticles	Franz diffusion cells and mice skin	Lecithin-chitosan nanoparticles enhanced the penetration of quercetin and augmented its retention in the epidermis	[238]
Curcumin	Microemulsion	HaCaT cells; Human skin	Substantial amounts of curcumin were detected in the dermis, and the application of curcumin microemulsion reduced the harmful effects of UV radiation on the outer layer of the skin	[239]
	Phytovesicles	Mice	The phytovesicles demonstrated superior efficacy in comparison to all other preparations and ordinary curcumin in delivering heightened antioxidant and anti-aging benefits	[240]
	Liposome (DLs) nanocarriers	Isolated human skin	Consistently infiltrated the skin and improved its biological characteristics	[241]
	Transparent plastid nanovesicles	Human keratinocytes	In vitro, it provided protection to human keratinocytes against damage caused by oxidative stress, mitigated inflammation and injury induced by 12-0-tetracyl-chlorowave, decreased edema development, and enhanced the biocompatibility and safety of the components	[242]
	Peptide-modified curcumin-loaded liposome (CRC-TD-Lip)	Mice	It demonstrated exceptional stability and a remarkable capacity to encapsulate curcumin, resulting in an expedited transdermal distribution of curcumin and an increased suppression of psoriasis	[243]

## Data Availability

Not applicable.
